# Comparison of prevalence of metabolic syndrome in hospital and community-based Japanese patients with schizophrenia

**DOI:** 10.1186/1744-859X-10-21

**Published:** 2011-09-12

**Authors:** Norio Sugawara, Norio Yasui-Furukori, Yasushi Sato, Ikuko Kishida, Hakuei Yamashita, Manabu Saito, Hanako Furukori, Taku Nakagami, Mitsunori Hatakeyama, Sunao Kaneko

**Affiliations:** 1 Department of Psychiatry, Hirosaki-Aiseikai Hospital, Hirosaki, Japan; 2 Department of Neuropsychiatry, Hirosaki University School of Medicine, Hirosaki, Japan; 3 Department of Psychiatry, Fujisawa Hospital, Fujisawa, Japan; 4 Department of Psychiatry, Yokohama City University School of Medicine, Yokohama, Japan; 5 Department of Psychiatry, Moro Hospital, Moroyama, Japan; 6 Department of Neuropsychiatry, Saitama Medical University, Moroyama, Japan; 7 Department of Psychiatry, Kuroishi-Akebono Hospital, Kuroishi, Japan; 8 Department of Psychiatry, Odate Municipal General Hospital, Odate, Japan; 9 Department of Psychiatry, Higashidai Hospital, Odate, Japan

## Abstract

**Background:**

Lifestyle factors, such as an unbalanced diet and lack of physical activity, may affect the prevalence of metabolic syndrome (MetS) in schizophrenic patients. The aim of this study was to compare the MetS prevalence between inpatients and outpatients among schizophrenic population in Japan.

**Methods:**

We recruited inpatients (n = 759) and outpatients (n = 427) with a *Diagnostic and Statistical Manual of Mental Disorders*, fourth edition (DSM-IV) diagnosis of schizophrenia or schizoaffective disorder from 7 psychiatric hospitals using a cross-sectional design. MetS prevalence was assessed using three different definitions, including the adapted National Cholesterol Education Program Adult Treatment Panel (ATP III-A).

**Results:**

The overall MetS prevalences based on the ATP III-A definition were 15.8% in inpatients and 48.1% in outpatients. In a logistic regression model with age and body mass index as covariates, being a schizophrenic outpatient, compared to being a schizophrenic inpatient, was a significant independent factor (odds ratio = 3.66 for males, 2.48 for females) in the development of MetS under the ATP III-A definition. The difference in MetS prevalence between inpatients and outpatients was observed for all age groups in males and for females over 40 years of age.

**Conclusions:**

Outpatients with schizophrenia or schizoaffective disorder in Japan had a high prevalence of MetS compared to inpatients. MetS in schizophrenic outpatients should be carefully monitored to minimize the risks. A change of lifestyle might improve MetS in schizophrenic patients.

## Introduction

A high prevalence of metabolic syndrome (MetS) has been reported among schizophrenic patients [1-3]. MetS has been related to an increased risk for cardiovascular diseases [4,5], diabetes [6] and mortality [7] and is defined as a cluster of metabolic disturbances including abdominal obesity, atherogenic dyslipidemia, hypertension and hyperglycemia [8].

Commonly used definitions for MetS are the National Cholesterol Education Program Adult Treatment Panel (NCEP ATP III) MetS definition [7] and the adapted NCEP ATP III (ATP III-A) definition, proposed by the American Heart Association (AHA) following the American Diabetes Association's (ADA's) lowering of the threshold for impaired fasting glucose to 100 mg/dl [9]. Because abdominal obesity is widely recognized as a measure of metabolic abnormality, the International Diabetes Federation (IDF) established a definition that stressed the importance of waist circumference [10]. However, the small physique of the Asian population made it difficult to use the same waist circumference criterion determined for those of European descent [11]. Therefore, modified criteria for waist circumference (90 cm for males and 80 cm for females) have been proposed for Asians in the ATP III-A [12] and IDF [13] definitions. In addition, a definition established by the Japan Society for the Study of Obesity (JASSO) [14] was also used in this study. Based on an area of 100 cm^2 ^of intra-abdominal fat, the cut-off value for waist circumference is 85 cm for males and 90 cm for females under the JASSO definition [15]. Although the Japanese Committee of the Criteria for Metabolic Syndrome established the JASSO definition, there has been controversy concerning the effective cut-off value for waist circumference [16].

Lifestyle factors, such as an unbalanced diet and lack of physical activity, could cause MetS. Patients with schizophrenia are at risk for developing obesity due to poor dietary habits or limited physical activity because of the negative symptoms of schizophrenia. In addition, Japan has the highest number of psychiatric beds per 100,000 people in the world [17]. The mean length of hospital stay is about 1.5 years [18]. Because schizophrenic inpatients have received controlled meals and occupational therapy, the lifestyles of schizophrenic patients may be different from those of outpatients.

To clarify the effect of environmental factors on MetS in the schizophrenic population, we compared the prevalence of MetS based on the type of care (inpatient vs outpatient). To the best of our knowledge, this is the first study carried out in the schizophrenic population.

## Methods

### Participants

This study was conducted between January 2007 and December 2008. Subjects were 759 inpatients (355 males and 404 females) and 427 outpatients (215 males and 212 females) from 7 psychiatric hospitals in Japan who were diagnosed with either schizophrenia or schizoaffective disorder based on the Diagnostic and Statistical Manual of Mental Disorders, fourth edition (DSM-IV) diagnosis. The diagnoses of the patients were recorded based on their medical charts. All subjects were previously instructed to fast from midnight prior to the assessment day. The data collection for this study was approved by the Ethics Committee of the Hirosaki University School of Medicine and all subjects provided written informed consent before participating in this study. The characteristics of the study population have been reported previously [19]. In this study, we reanalyzed the subjects based on the type of care (inpatient vs outpatient).

### Measurements

The subjects' demographic data (age and sex) were obtained from their medical records. The height and weight of the subjects were measured, and body mass index (BMI) was calculated. Waist circumference to the nearest 0.1 cm was measured at the umbilical level with the subject in a standing position by a technician in the morning. Trained technicians measured blood pressure (BP) using standard mercury sphygmomanometers on the right arm of seated participants after a 5 min rest period. High-density lipoprotein (HDL) cholesterol, triglycerides and fasting blood glucose were also measured using standard analytical techniques. The presence of MetS was determined based on the definitions given by the ATP III-A for Asians, the recent IDF for Japanese populations and the JASSO (Table 1).

**Table 1:** Definitions of metabolic syndrome

	ATP III-A^a^	IDF^b^	JASSO^c^
Waist circumference (cm)	Male ≥ 90, female ≥ 80	Male ≥ 90, female ≥ 80	Male ≥ 85, female ≥ 90
Blood pressure (mmHg)^d^	≥ 130/85	≥ 130/85	≥ 130/85
HDL (mg/dl)^e^	Male < 40, female < 50	Male < 40, female < 50	< 40
TG (mg/dl)^e^	≥ 150	≥ 150	≥ 150
Glucose (mg/dl)^f^	≥ 100	≥ 100	≥ 110

^a^Metabolic syndrome if three of five criteria are met.

^b^Metabolic syndrome if waist circumference plus two criteria are met.

^c^Metabolic syndrome if waist circumference plus two of the following criteria are met: high blood pressure, reduced high-density lipoprotein (HDL) and/or raised triglyceride (TG), raised fasting hyperglycemia.

^d^Or specific treatment of previously diagnosed hypertension.

^e^Or specific treatment for this lipid abnormality.

^f^Or specific treatment with insulin or hypoglycemic medication.

ATP III-A = Adapted National Cholesterol Education Program Adult Treatment Panel; IDF = International Diabetes Federation; JASSO = Japan Society for the Study of Obesity.

### Statistical analysis

Descriptive statistics were computed to describe the demographic and clinical variables. In order to compare the main demographic and clinical characteristics between groups, the unpaired Student's t test was performed to analyze continuous variables, and a χ^2 ^test or Fisher's exact test was performed to analyze categorical variables. After adjusting for confounding factors (age and BMI), a multivariate logistic regression analysis was performed to assess the influence of schizophrenia as a risk factor for MetS. A value of *P *< 0.05 was considered significant. The data were analyzed using SPSS software for Windows (Version 12.0).

## Results

### Demographic and clinical characteristics

Demographic and clinical characteristics of the study population are shown in Table 2. Schizophrenic outpatients were significantly younger and taller, and had higher weight, BMI, waist circumference, systolic BP, diastolic BP, triglyceride and fasting blood glucose than schizophrenic inpatients.

**Table 2:** Demographic and clinical characteristics of the subjects

	Total			Male			Female		
	
	Inpatients, (n = 759)	Outpatients, (n = 427)	*P *value	Inpatients, (n = 355)	Outpatients, (n = 215)	*P *value	Inpatients, (n = 404)	Outpatients, (n = 212)	*P *value
Age (years)	59.9 ± 12.9	45.6 ± 13.6	< 0.001	58.3 ± 13.1	45.2 ± 13.5	< 0.001	61.4 ± 12.5	46.0 ± 13.7	< 0.001
Height (cm)	158.3 ± 9.8	162.6 ± 9.9	< 0.001	165.1 ± 7.2	168.5 ± 6.6	< 0.001	152.3 ± 7.7	156.4 ± 8.9	< 0.001
Weight (kg)	55.7 ± 11.8	69.3 ± 14.4	< 0.001	60.8 ± 11.6	75.2 ± 11.9	< 0.001	51.2 ± 10.1	63.2 ± 14.2	< 0.001
BMI (kg/m^2^)	22.1 ± 4.0	26.5 ± 11.4	< 0.001	22.2 ± 4.0	26.5 ± 3.9	< 0.001	22.0 ± 4.0	26.6 ± 15.7	< 0.001
Waist circumference (cm)	82.9 ± 10.7	90.3 ± 12.4	< 0.001	82.9 ± 10.3	92.8 ± 10.7	< 0.001	83.0 ± 11.0	87.5 ± 13.4	< 0.05
Systolic BP (mmHg)	117.8 ± 16.1	127.9 ± 20.0	< 0.001	119.6 ± 16.5	131.4 ± 19.8	< 0.001	116.2 ± 15.5	124.4 ± 18.8	< 0.001
Diastolic BP (mmHg)	73.3 ± 11.6	78.7 ± 13.1	< 0.001	75.2 ± 12.1	80.9 ± 12.9	< 0.001	71.7 ± 10.8	76.5 ± 13.0	< 0.001
HDL-C (mg/dl)	55.0 ± 15.3	53.8 ± 16.1	NS	49.8 ± 12.5	49.3 ± 15.3	NS	59.5 ± 16.1	58.4 ± 15.6	NS
Triglyceride (mg/dl)	96.7 ± 56.5	167.8 ± 126.1	< 0.001	99.1 ± 63.6	202.3 ± 148.4	< 0.001	94.6 ± 49.5	133.2 ± 86.2	< 0.001
Fasting glucose (mg/dl)	90.6 ± 18.6	115.7 ± 52.2	< 0.001	91.0 ± 20.9	118.1 ± 53.2	< 0.001	90.3 ± 16.3	113.3 ± 51.2	< 0.001

Data are expressed as mean ± SD. These data were analyzed using Student's t test between the reference group and the schizophrenic patients.

BP = blood pressure; BMI = body mass index; HDL-C = high-density lipoprotein cholesterol; NS = not significant.

### MetS and criteria prevalence among subjects

The data in Table 3 shows significant patterns of MetS criteria prevalence by type of care (inpatients vs outpatients). The unadjusted MetS prevalences among schizophrenic outpatients (inpatients) using the ATP III-A, IDF and JASSO definitions were 48.1% (15.8%), 44.1% (14.7%) and 33.2% (9.1%), respectively. The prevalence of all the criteria is significantly higher in the outpatient group than in the inpatient group. Among schizophrenic inpatients, an association between gender and MetS prevalence was significant based on the JASSO and IDF definitions (JASSO: χ^2 ^= 16.03, df = 1, *P *< 0.001, IDF: χ^2 ^= 4.04, df = 1, *P *< 0.05) but was not significant based on the ATP III-A definition (χ^2 ^= 1.49, df = 1, *P *= 0.22). Schizophrenic outpatients showed an association between gender and MetS prevalence that was significant based on the JASSO and ATP III-A definitions (JASSO: χ^2 ^= 31.19, df = 1, *P *< 0.001, ATP III-A: χ^2 ^= 9.94, df = 1, *P *< 0.01), but was not significant based on the IDF definition (χ^2 ^= 3.60, df = 1, *P *= 0.058).

**Table 3:** Prevalence of metabolic syndrome (MetS) and its criteria among subjects

	All			Male			Female		
	
	Inpatients	Outpatients	*P *value	Inpatients	Outpatients	*P *value	Inpatients	Outpatients	*P *value
MetS prevalence:									
ATP III-A	15.8	48.1	< 0.001	14.1	55.8	< 0.001	17.3	40.6	< 0.001
IDF	14.7	44.1	< 0.001	11.9	48.6	< 0.001	17.1	39.4	< 0.001
JASSO	9.1	33.2	< 0.001	13.6	45.8	< 0.001	5.1	20.2	< 0.001
MetS criteria prevalence:									
Waist circumference									
Male ≥ 90 cm, female ≥ 80 cm	46.2	64.2	< 0.001	27.8	61.7	< 0.001	62.4	66.8	NS
Male ≥ 85 cm, female ≥ 90 cm	34.9	59.0	< 0.001	42.3	73.8	< 0.001	28.4	43.8	< 0.001
BP (≥ 130/85 mmHg)	30.7	46.7	< 0.001	37.2	55.5	< 0.001	25.1	37.5	< 0.01
HDL (male < 40 mg/dl, female < 50 mg/dl)	25.5	31.8	< 0.05	22.7	28.9	NS	28.0	34.8	NS
Triglyceride (≥ 150 mg/dl)	11.2	40.6	< 0.001	13.2	50.2	< 0.001	9.4	31.0	< 0.001
HDL (< 40 mg/dl) and TG (≥ 150 mg/dl)	20.8	46.1	< 0.001	28.2	58.1	< 0.001	14.4	34.0	< 0.001
Glucose (≥ 100 mg/dl)	17.5	50.9	< 0.001	16.6	56.0	< 0.001	18.4	45.5	< 0.001
Glucose (≥ 110 mg/dl)	9.5	35.3	< 0.001	9.9	36.8	< 0.001	9.2	33.7	< 0.001

All prevalences are expressed as a percentage (%) and were analyzed using the χ^2 ^test comparing the reference group and the schizophrenic patients.

ATP III-A = Adapted National Cholesterol Education Program Adult Treatment Panel; BP = blood pressure; IDF = International Diabetes Federation; JASSO = Japan Society for the Study of Obesity; HDL = high-density lipoprotein; NS = not significant.

Male inpatients with schizophrenia showed higher prevalences of criteria for waist circumference (JASSO: χ^2 ^= 15.65, df = 1, *P *< 0.001), BP (ATP III-A, IDF, JASSO: χ^2 ^= 12.98, df = 1, *P *< 0.001) and TG and/or HDL (JASSO: χ^2 ^= 21.71, df = 1, *P *< 0.001) than female inpatients. However, more female inpatients with schizophrenia met the criteria for waist circumference (ATP III-A, IDF: χ^2 ^= 88.17, df = 1, *P *< 0.001) than male inpatients. No significant differences were seen in HDL (ATP III-A, IDF: χ^2 ^= 2.69, df = 1, *P *= 0.10), TG (ATP III-A, IDF: χ^2 ^= 2.75, df = 1, *P *= 0.097) and fasting plasma glucose levels (ATP III-A, IDF: χ^2 ^= 3.96, df = 1, *P *= 0.529, JASSO: χ^2 ^= 0.10, df = 1, *P *= 0.751).

Male outpatients with schizophrenia showed higher prevalences of criteria for waist circumference (JASSO: χ^2 ^= 39.46, df = 1, *P *< 0.001), BP (ATP III-A, IDF, JASSO: χ^2 ^= 13.57, df = 1, *P *< 0.001), TG (ATP III-A, IDF: χ^2 ^= 15.53, df = 1, *P *< 0.001), TG and/or HDL (JASSO: χ^2 ^= 23.80, df = 1, *P *< 0.001) and fasting plasma glucose levels (ATP III-A, IDF: χ^2 ^= 4.48, df = 1, *P *< 0.05) than female outpatients. No significant difference was seen in waist circumference (ATP III-A, IDF: χ^2 ^= 1.22, df = 1, *P *= 0.270), HDL (ATP III-A, IDF: χ^2 ^= 1.49, df = 1, *P *= 0.22) and fasting plasma glucose levels (JASSO: χ^2 ^= 0.46, df = 1, *P *= 0.50)

### The effect of type of care on the odds ratio for MetS

To examine the independent effect of type of care for schizophrenia on the odds ratio of for MetS, two logistic regression models were developed with MetS status as the binary dependent variable (Table 4). Due to the differences in the criteria for MetS by gender, these models were constructed in a gender-specific manner. In model 1, the odds ratios of having MetS were greater for male schizophrenic outpatients (ATP III-A: odds ratio = 7.57, 95% CI = 4.83 to 11.86, *P *< 0.001, IDF: odds ratio = 6.72, 95% CI = 4.19 to 10.78, *P *< 0.001, JASSO: odds ratio = 6.07, 95% CI = 3.80 to 9.71, *P *< 0.001), and female schizophrenic outpatients (ATP III-A: odds ratio = 4.24, 95% CI = 2.70 to 6.67, *P *< 0.001, IDF: odds ratio = 3.95, 95% CI = 2.50 to 6.24, *P *< 0.001, JASSO: odds ratio = 5.66, 95% CI = 2.92 to 10.94, *P *< 0.001) when analyzed with illness and age as covariates. In the second model, the odds ratios for both male and female schizophrenic outpatients were also statistically significant when BMI was added as a covariate.

**Table 4:** Logistic regression models of metabolic syndrome (MetS) status in subjects

	Model 1			Model 2		
	
	Covariate	OR (95% CI)	*P *value	Covariate	OR (95% CI)	*P *value
Male:						
ATP III-A	Outpatients	7.569 (4.829 to 11.863)	< 0.001	Outpatients	3.664 (2.187 to 6.139)	< 0.001
	Age	1.000 (0.985 to 1.015)	NS	Age	1.015 (0.998 to 1.036)	NS
				BMI	1.479 (1.365 to 1.602)	< 0.001
IDF	Outpatients	6.717 (4.188 to 10.7765)	< 0.001	Outpatients	2.506 (1.397 to 4.496)	< 0.01
	Age	0.998 (0.983 to 1.014)	NS	Age	1.018 (0.996 to 1.040)	NS
				BMI	1.677 (1.517 to 1.855)	< 0.001
JASSO	Outpatients	6.071 (3.795 to 9.711)	< 0.001	Outpatients	2.599 (1.505 to 4.488)	< 0.01
	Age	1.011 (0.995 to 1.027)	NS	Age	1.034 (1.014 to 1.055)	< 0.01
				BMI	1.467 (1.354 to 1.589)	< 0.001
Female:						
ATP III-A	Outpatients	4.243 (2.699 to 6.671)	< 0.001	Outpatients	2.477 (1.474 to 4.161)	< 0.01
	Age	1.017 (1.002 to 1.032)	< 0.05	Age	1.032 (1.013 to 1.050)	< 0.01
				BMI	1.332 (1.253 to 1.416)	< 0.001
IDF	Outpatients	3.952 (2.497 to 6.254)	< 0.001	Outpatients	2.842 (1.738 to 4.647)	< 0.001
	Age	1.015 (1.000 to 1.030)	NS	Age	1.021 (1.004 to 1.038)	< 0.05
				BMI	1.143 (1.090 to 1.198)	< 0.001
JASSO	Outpatients	5.655 (2.923 to 10.940)	< 0.001	Outpatients	4.137 (2.047 to 8.361)	< 0.001
	Age	1.010 (0.989 to 1.031)	NS	Age	1.012 (0.990 to 1.035)	NS
				BMI	1.089 (1.027 to 1.154)	< 0.01

ATP III-A = Adapted National Cholesterol Education Program Adult Treatment Panel; BMI = body mass index; IDF = International Diabetes Federation; JASSO = Japan Society for the Study of Obesity; OR = odds ratio; NS = not significant.

### Age-specific prevalence of metabolic syndrome

**Figure 1:**
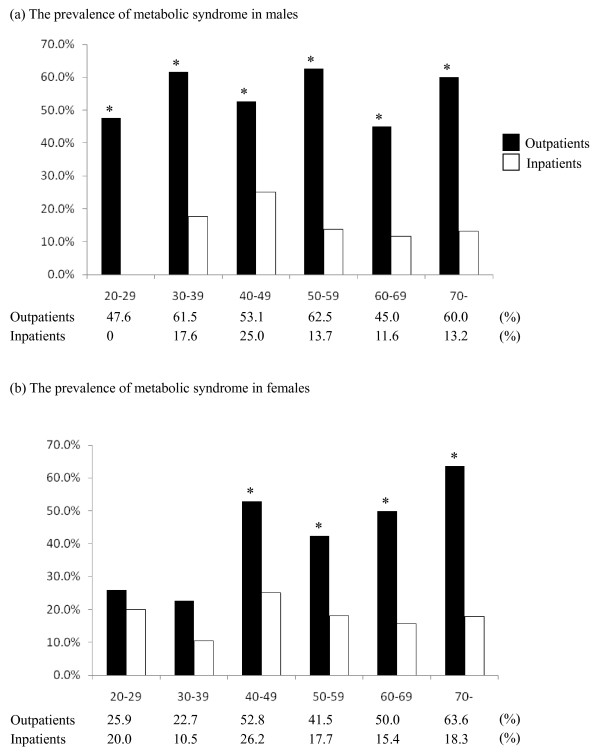
**The age-specific prevalence of metabolic syndrome (Adapted National Cholesterol Education Program Adult Treatment Panel (ATP III-A)) among Japanese outpatients and inpatients with schizophrenia**. *Indicates a statistically significant (*P *< 0.05) difference from control.

Figure 1 shows the age-specific prevalences of MetS (ATP III-A) for both genders. In all age groups, MetS prevalence for male schizophrenic outpatients was greater than inpatients. For female schizophrenic outpatients, the prevalence was statistically higher in the over 40 age group. The age-specific prevalences of MetS using the IDF and JASSO definitions showed similar tendencies (data not shown).

## Discussion

Control of diet and physical activity may affect the development of MetS. The services provided for patients differ based on the type of care, such as hospital or community-based care. The type of care could be a large environmental factor. However, there have been few studies that compare the prevalence of MetS among patients receiving different types of care. In this study, we reported the prevalence of MetS in inpatients and outpatients diagnosed with schizophrenia. Compared to inpatients, outpatients were found to be at a higher risk of developing MetS.

Previous studies of inpatients with schizophrenia have reported that the prevalence of MetS ranged from 27 to 29% using the ATP III definition [20,21]. With the same definition, the prevalence of MetS among outpatients with schizophrenia ranged from 25% to 35% [3,22]. Although there have been some reports of the prevalence of MetS in patients undergoing inpatients or outpatient care, we could not compare the prevalence of MetS because the patients in these studies were treated under different systems of medical care.

In this study, outpatients with schizophrenia were found to be at a higher risk of developing MetS than inpatients with schizophrenia. Using the ATP III-A and JASSO definitions, male outpatients have a higher prevalence of MetS than female outpatients. Among inpatients with schizophrenia, female inpatients showed a higher prevalence of MetS than male inpatients using the IDF definition, while male inpatients showed a higher prevalence of MetS than female inpatients using the JASSO definition.

The prevalence rates of MetS reported in previous studies have varied considerably due to the different definitions of the syndrome used in each study. In this study, the prevalence rate of MetS (5.1%) among female schizophrenic inpatients using the JASSO definition seems to be extremely low compared to those obtained using the ATP III-A (17.3%) and IDF (17.1%) definitions. This difference may be due to the influence of increasing the waist circumference criterion to 90 cm [14]. The specific values of waist circumference used to assess MetS in the Japanese population have changed in the IDF definition: the current specific values for Japanese are 90 cm for men and 80 cm for women [13], as in the ATP III-A definition. Though the effective cut-off value for waist circumference is still controversial, the ATP III-A or IDF criteria may be suitable for making international comparisons because of the availability of data in several different ethnic groups.

As mentioned earlier, no significant difference in MetS prevalence between inpatients and outpatients was observed in females aged 39 years and younger. The results using the IDF and JASSO definitions were similar. One possible explanation is that this study failed to find a difference in MetS prevalence in females aged 39 years and younger due to smaller sample sizes. Another possible explanation is that the later age at onset of female schizophrenic patients compared to schizophrenic male patients [23,24] might cause differences in duration of treatment between genders.

The reason for this increased prevalence among outpatients has not been entirely elucidated. However, schizophrenic inpatients have received controlled diets and occupational therapy. Schizophrenic inpatients in Japan typically have long hospital stays, and the above-mentioned treatment might influence the lower prevalences of MetS in this study. Previous studies [25,26] have suggested that long-term programs that incorporate nutrition, exercise, and behavioral interventions can prevent weight gain among schizophrenic patients. An effective intervention program could reduce the high risk for developing MetS among male outpatients.

The current study also has some limitations. Firstly, it was a cross-sectional study. It is necessary to carry out a follow-up survey to clarify the reason for not finding a difference in the MetS prevalence between inpatients and outpatients among female subjects aged 39 and younger. Secondly, patient recruitment was restricted to hospitals where outpatients and inpatients presented for a review of their health problems. No other population groups were included, such as children, adolescents or unmedicated patients. Thirdly, some patients, who were diagnosed with schizophrenia may have had metabolic disturbances prior the use of antipsychotics [27]. Lastly, some parameters that may contribute to MetS were not included in this study, such as dietary habits, physical activity levels, duration of illness and treatment, length of the current stay in hospital among inpatients, schizophrenic symptoms and medications. Antipsychotic medications may be especially important factors. The use of first-generation or second-generation antipsychotics might confound the results. Stratification by drug use is needed in further studies.

## Conclusions

This study has shown that the prevalence of MetS in Japanese outpatients with schizophrenic and schizoaffective disorders was higher than in inpatients and was considerably higher in male outpatients. Therefore, metabolic abnormalities in schizophrenic patients should be monitored carefully and treated in an appropriate manner.

## Competing interests

The authors declare that they have no competing interests.

## Authors' contributions

NS conceived the study, designed the study, conducted the statistical analysis, interpreted the data and wrote the initial draft of the manuscript. SK had full access to all of the data in the study and takes responsibility for the integrity of the data and the accuracy of the data analysis. SK and NYF contributed to study design and assisted in drafting the manuscript. YS, IK and HY completed the initial survey construction and recruitment of participants. MS, HF, TN and MH participated in the data collection, and the interpretation of the results. All authors have approved the manuscript.
